# Supplementary material for: Dementia- and mild cognitive impairment-inclusive exercise: Perceptions, experiences, and needs of community exercise providers
Source: PLoS One. 2020 Sep 8;15(9):e0238187. doi: 10.1371/journal.pone.0238187 (PMC7478809; doi:10.1371/journal.pone.0238187)
Supplement: S1 File — (DOCX) [file pone.0238187.s001.docx]

| *Introduction/warm-up question:* State your name, current role as an exercise facilitator and experience with dementia in general, whether it be personal or professional.   1. What comes to mind when you think about dementia and mild cognitive impairment (MCI)?  - *How would you recognize that someone you were training was likely to have dementia or cognitive impairment?*  1. What comes to mind when thinking about people with dementia and MCI and exercising?  - *What are the benefits? Risks?* - *What challenges have you experienced or could you foresee when working with people with MCI or dementia?* - *What strategies would you use/have you used? What worked and what didn't work?*  1. What do you feel you need in order to provide the best services to clients with dementia or MCI? For example, training, support, knowledge or other resources?  - *Is there anything in particular that worries or concerns you about working with people with dementia and MCI?* - *Is there anything that has helped prepare you or current resources you know of?* - *What do you feel is missing or necessary?* - *How would you like to get training/information/resources?*  1. What are your thoughts on including people with MCI/dementia into the existing exercise programs at your site, particularly those not designed specifically for people with MCI/dementia?  - *What supports/knowledge/resources would you need to make it feasible?* - *What are the challenges/benefits of inclusive programs?* - *What would be the reaction of other participants?* - *What would be the role of the care partner?*  1. What are your thoughts on running dementia/MCI specific programs at your site?    - *What might be the challenges with a dementia specific program?*    - *What might be the benefits of a dementia specific program?*    - *What supports and resources would you need to make a dementia specific program happen?*    - *What would be the role of the care partner?* 2. What are your thoughts on the idea of a home-based program for people with dementia and MCI?  - *What might a home-based program look like and what would be needed to make it effective?* - *What would be the benefits of a home-based program be? What would be the risks or disadvantages?* - *Have you ever prescribed/given exercises to clients you suspect may have cognitive impairment to do at home? If so, what was your experience with this?* - *What would be the role of the care partner?*   *Closing Question:* From our discussion, what do you think was an interesting point brought up or the take-home message? |
| --- |
