# Supplementary material for: Dementia- and mild cognitive impairment-inclusive exercise: Perceptions, experiences, and needs of community exercise providers
Source: PLoS One. 2020 Sep 8;15(9):e0238187. doi: 10.1371/journal.pone.0238187 (PMC7478809; doi:10.1371/journal.pone.0238187)
Supplement: Attachment — Submitted filename: Response to Reviewers - 2020-08-05.docx [file pone.0238187.s002.docx]

**Response to Reviewers – Manuscript PONE-D-20-0999**

Thank-you for your prompt response to the revisions that were submitted. Responses to the editor’s comments are provided below and are reflected in the updated manuscript submissions.

**Editor’s comments**

Line 216 and elsewhere – please change ‘professional designations’ to ‘professional qualifications’ or ‘professional backgrounds’

- This has been changed to qualifications / backgrounds as appropriate in Line 216 and elsewhere.

Remove “ “ from “other”

- This has been removed in Line 217.

Please reword sentence now on line 241 from ‘tended more to’ to “were more likely to” – ‘tended more to’ is very clunky phrasing and in the context of a qual study ‘more likely to’ will not be misinterpreted as inferring statistical likelihood

- This has been reworded in Line 241.
